# Introduction to Artificial Intelligence for General Surgeons: A Narrative Review

**DOI:** 10.7759/cureus.79871

**Published:** 2025-03-01

**Authors:** Blanche Lee, Nikhil Narsey

**Affiliations:** 1 General Surgery, Toowoomba Hospital, Toowoomba City, AUS

**Keywords:** acute trauma care, artificial intelligence in surgery, computer vision, deep learning artificial intelligence, machine learning models, natural language processing (nlp)

## Abstract

Artificial intelligence (AI) has rapidly progressed in the last decade and will inevitably become incorporated into trauma and surgical systems. In such settings, surgeons often need to make high-stakes, time-sensitive, and complex decisions with limited or uncertain information. AI has great potential to augment the pre-operative, intra-operative, and post-operative phases of trauma care. Despite the expeditious advancement of AI, many surgeons lack a foundational understanding of AI terminology, its processes, and potential applications in clinical practice. This narrative review aims to educate general surgeons about the basics of AI, highlight its applications in thoraco-abdominal trauma, and discuss the implications of incorporating its use into the Australian health care system. This review found that studies of AI in trauma care have predominantly focused on machine learning and deep learning applied to diagnostics, risk prediction, and decision-making. Other subfields of AI include natural language processing and computer vision. While AI tools have many potential applications in trauma care, current clinical use is limited. Future prospective, locally validated research is required prior to incorporating AI into clinical practice.

## Introduction and background

Artificial intelligence (AI) is rapidly evolving in the 21st century and powering the Fourth Industrial Revolution [1]. This is made possible as global funding into the AI sector surges, exponential advances are made in computational power and data storage, and as the digitalisation of data in hospitals with electronic medical records (EMR) produce massive datasets [2,3]. AI can be broadly defined as technology that enables machines to perform human-like tasks such as image processing, problem-solving, decision-making, and understanding language. Four subfields of AI include machine learning (ML), computer vision (CV), natural language processing (NLP), and deep learning (DL), also known as deep neural networks (DNNs) [4]. AI marks the intersection between mathematics, statistics, computer and data sciences, linguistics, hardware and software engineering, cognitive science, ethics, and philosophy. AI is positioned to make an exigent entry into the healthcare sector and will inevitably be integrated into the future of medical and surgical services. Currently however, Australia significantly lags behind the rest of the world with respect to funding, research, and clinical applications of AI [5,6]. A *Lancet* scoping review identified 86 randomised controlled trials (RCTs) on AI performed worldwide which were published between 2018 and 2023: none were performed in Australia [7]. While AI technology has been extensively assessed in imaging sectors such as radiology, its adoption in surgery has been slow [8]. Many surgeons are aware of the concept of AI but lack specific knowledge and practical experience of its use. A global survey found general scepticism from surgeons on the use of AI to support clinical decision-making in emergency and trauma surgery [9]. Many surgeons conveyed a low degree of acceptance of AI, and a majority demonstrated a limited understanding of AI applications in surgery [9]. Surgeons preferred classical decision-making aids such as traditional training, clinical guidelines, and multidisciplinary committees [9]. A key concern may lie in the lack of technical understanding of AI, and lack of clarity on legal responsibility of its use. Another international survey of surgeons on their self-rated knowledge of AI terminology found that 24% were unable to define any AI terminology, and 50% were only able to partially define AI concepts such as narrow and general AI, ML, DL, supervised and unsupervised learning, CV, and NLP [10]. The low survey response rate of 2% from members of the World Society of Emergency Surgery increases the risk of selection bias where those interested in AI are more likely to respond [10]. As global AI innovation continues, it is imperative for surgeons to gain a basic understanding of AI. AI terminology can be unfamiliar to surgeons who have received limited or no education on this topic during medical training. The purpose of this narrative review is to educate surgeons about the basics of AI terminology, its clinical applications and implications in thoraco-abdominal trauma, and highlight the potential for those algorithms to transform surgical care in the future.

Introduction to AI 

**Figure 1:**
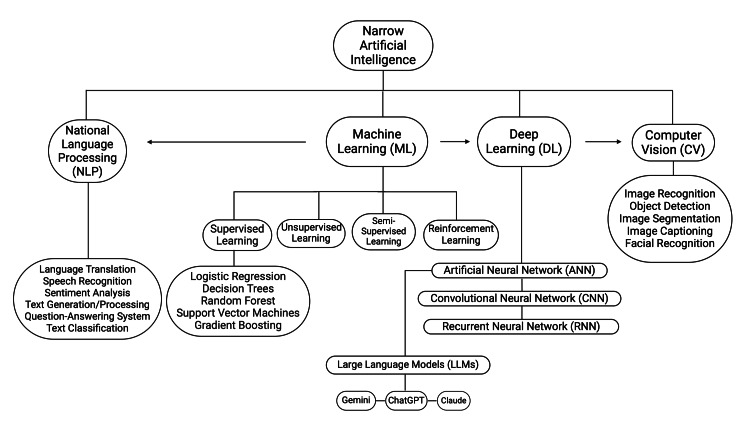
Narrow AI Taxonomy

The term 'artificial intelligence' was coined in 1955 by Stanford Professor McCarthy as “the science and engineering of making intelligent machines” [2]. Most AI applications in use fall under the category of narrow AI. This is defined as an intelligent system designed to perform a single task such as image recognition or translation of language. In contrast, artificial general intelligence refers to an intelligent system which aims to perform actions that mimic human general intelligence, self-learns and reasons, and has human-like cognitive abilities [2]. Figure 1 provides an overview of narrow AI taxonomy.

Machine learning

ML is a subfield of AI and uses algorithms to imitate the way humans learn. ML algorithms can make a prediction or classification by making an estimate about patterns in data. The model will evaluate the prediction and improve its accuracy through an iterative optimisation process [11]. ML can be further classified into supervised (labelled data), unsupervised (unlabelled data), and semi-supervised (labelled and un-unlabelled data). Reinforcement learning is another branch of ML where decision-making is performed by an autonomous agent such as a robot or self-driving car through an interaction between an agent, environment and goal [12]. Within trauma medicine, supervised ML algorithms are the most studied in published literature [13]. Supervised ML is more dependent on human intervention to learn because the input data is labelled, and the model delivers a predefined output. Supervised ML algorithms include logistic regression, decision trees, random forest, support vector machines (SVM), and gradient boosting. The receiver-operating characteristic (ROC) curve is drawn by calculating the true-positive rate (TPR) and false-positive rate (FPR) at selected intervals, and then the TPR is graphed over the FPR [14]. A perfect model at a certain threshold would have a TPR of one and a FPR of zero. Generally, the model with a greater area under the ROC curve (AUROC) is the better one, and this can help compare the performance of two different models if the dataset is balanced.

Deep learning

DL is a subset of ML and is advantageous in that it can be scalable to large data sets. DL can use labelled, unlabelled, or unstructured data in raw forms (e.g. text or images) to develop its own representation for pattern recognition [3,11]. The ability to use unstructured data is particularly useful in a healthcare setting where data is often heterogenous in nature [3]. DL uses neural networks, similar to the neuronal connections in the human brain, to make complex decisions. Other DL networks include recurrent neural networks (RNNs) and convolutional neural networks (CNNs). DL differs from traditional ‘non-deep’ ML in that it utilises three or more computational layers, normally hundreds or thousands, to train the models. Neural networks are comprised of node layers including an input, one or more hidden layers, and an output layer. These node layers are connected to each other via ‘interneurons’ and each hold a threshold value and weight [15]. The input and output node layers are termed ‘visible’ layers. Computational progression through the layers can occur via forward or backpropagation to enable the algorithm to predict and correct for errors. With time, the model become more accurate as it learns. While DL offers many advantages, it requires significant computational power to train algorithms and is costly to scale [15].

Computer vision

CV is a subfield of DL which uses computers and systems to understand digital images and videos, and then acts or make a recommendation based upon these inputs. CV enable computers to see and observe through image detection, object classification, and segmentation. CV utilises a type of DL called CNNs to perform these tasks. While it has greater scalability to large data sets, it is also computationally demanding, requires graphical processing units, and necessitates a vast amount of data to train itself. CV has particularly promising results in complex, image-heavy diagnostics fields relating to radiology, dermatology, ophthalmology, and pathology [3]. CV has also been used to study the intra-operative phase of care to augment surgical decision-making, support safer surgery, and expand access to surgical data [16]. However, there are currently no widely used CV tools in surgery.

Natural language processing

NLP enables computers and devices to recognise, understand, and generate text and speech by combining ML and DL with computational linguistics [17]. Unstructured text and voice data can be used to train large language models to perform tasks like language translation and sentiment analysis. NLP is commonly used to power search engines, chatbots, and voice-operated digital assistants on smartphones or global positioning system (GPS). The potential benefit of utilising NLP in a healthcare system with EMRs is a rapidly expanding area of research [3]. Within a single hospital admission, ~150,000 pieces of data are generated [3]. This data ranges from pathology results, imaging reports, ward round notes, and patient outcome data. Analysis of such data could provide significant diagnostic, prognostic, and decision-making support for clinicians.

## Review

AI in thoracic and abdominal trauma

AI has a wide range of potential applications in trauma systems in the pre-operative, intra-operative, and post-operative phases. However, this review found that AI tools remain largely conceptual and are almost non-existent in clinical settings in Australia [6]. AI-derived ML and DL tools can enhance diagnostics, predictive analytics, risk stratification, patient selection for operative intervention, and guide shared decision-making between the patient and the clinician, triage allocation of resources, and aid in discharge planning. Studies of CV tools remain preliminary but have the potential to enhance surgical education and training through augmented reality and real-time intra-operative assistance [16]. NLP tools could streamline documentation and communication, data collection and auditing, and inform patient consent and follow-up [18]. 

Pre-operative 

*Prediction of Haemorrhage Control* Zhang et al. evaluated various ML models in predicting the need for urgent haemorrhage control, which was defined as the activation of a massive transfusion protocol, endovascular embolisation, or damage control surgery [19]. Five ML models were compared, and data was collected from the United States National Trauma Data Bank. Seventeen input variables including vital signs, Glasgow Coma Scale (GCS), injury patterns to the head, neck, torso, abdomen, pelvis, and limbs were collected within four hours of the trauma incident. The XGBoost model performed the best across training and internal and external data validation sets. It achieved an AUROC of 0.88 with an accuracy of over 81% [19]. 

*Thoracic Trauma Imaging* Rib fractures are the most common sequelae of traumatic chest injury and result in significant morbidity and mortality. The rate of missed rib fractures by radiologists is up to 20.9% [20]. ML has been used to reduce clinical time for the detection of fractures in chest radiography and CT with promising results [20-22]. Wang et al. used DNNs to segment ribs and detect rib fractures with an AUROC of 0.94, sensitivity of 86.2%, and specificity of 98.8%, which was on par with attending radiologists [23]. AI misdiagnoses were significantly reduced when a radiologist reviewed the same images [21]. This highlights the utility of AI as a second-reader in trauma imaging and the central role a radiologist plays in verifying the accuracy of AI. Similar studies have been performed to study the use of ML in the detection of pulmonary contusions, pneumothorax, and hemothorax [24]. 

*Abdominal Trauma Imaging* DL demonstrates significant potential in the automated detection of visceral organ injury and lesions through image segmentation and volumetric calculations [25,26]. Cheng et al. used 1302 venous phase CT scans from a single trauma centre in Taiwan to train a model to detect intra-abdominal injury and tested it on 194 CT scans [26]. The spleen injury model had an AUROC of 0.95 and accuracy of 0.94, the liver model had an AUROC of 0.87 and accuracy of 0.82, and the kidney model had an AUROC of 0.87 and accuracy of 0.95. While the individual organ models demonstrated reasonably good accuracy, the whole image model lacked the capacity to identify specific injured organs reliably. Among 72 patients, 19 cases (26.4%) of solid organ injuries were missed [26]. Shen et al. trained a DL model to identify multiple organ injuries simultaneously with a database of 4000 CT images from 14 countries [25]. This model was trained to detect extravasation of contrast media to identify bleeding, as well as simultaneous detection of injuries to the liver, spleen, kidney, or bowel. The accuracy of the model was best for renal injuries with an AUROC of 0.88 and accuracy of 0.93. Liver injury detection had an AUROC of 0.82 and accuracy of 0.87, while splenic injury had an AUROC of 0.85 and an accuracy of 0.77. In contrast, bowel injuries had a low positive predictive value (PPV) and poor sensitivity despite having an AUROC of 0.83 and accuracy of 0.98. This suggests the model can be used to reliably rule out intestinal injury, but is poor at flagging positive cases [25]. Similarly, contrast blush could be reliably ruled out, but the model had low PPV and poor sensitivity.

*ICU Admission and Length of Stay* ML has been used to predict ICU admission and length of stay following thoraco-abdominal trauma using clinical parameters and CT imaging findings [27]. Clinical parameters included age, sex, vital signs, haemoglobin, haematocrit, Revised Trauma Score, GCS, and lactate. Imaging findings included the presence or absence of solid organ injury as per the American Association for the Surgery of Trauma Grading, intra-peritoneal fluid, thoracic aortic injuries, rib fractures, pelvic fractures, and haemothorax among other injuries. The AUROC of the SVM models for ICU admission and predicting length of stay was 0.87 and 0.80, respectively [27]. The predictions of outcomes were superior when combining clinical and CT imaging parameters.

Intra-operative

*Surgical Data Sciences* Surgery demands a high physical and cognitive load, and any error can translate to significant harm for patients. While the effectiveness of surgery has increased in the last few decades, there is still significant potential to improve surgical safety. Surgical Data Science has emerged as a novel discipline where digital data can be analysed and audited to improve surgical care [28]. Surgical big data incorporates studying and recording the operating room (OR) environment, intra-operative videos, surgical device logs, robot kinematics, patient’s physiological parameters, and measures of surgeon’s heart rate variability as a proxy of cognitive workload [28]. This vast amount of data can be analysed offline or in real-time with DL and ML algorithms to inform and enhance surgical care.

*Surgical Education, Training, and Feedback* The use of a surgical ‘black box’ and ‘surgical control towers’ has been proposed by Mascagni et al. as a method to systematically capture, analyse, and provide feedback to assist the operator [28]. These OR black boxes allow for post-operative analysis of near-misses or errors, and draw from parallels with their use in aviation and Formula One. The surgical black box is a multi-port device which captures synchronised audio-visual, environmental, and physiological data from the OR. Similarly, OR control towers, analogous to air traffic control, could live stream data in and out of the OR to permit real-time analysis and provide rapid feedback to the training or operating surgeon [28]. CV technology allows algorithms to interpret and analyse intra-operative visual data to support surgeon decision-making, enable safer surgery, and could democratise surgical care through international collaboration and education [16]. CV tools are still being refined in their ability to define intra-operative anatomy, tissue and dissection planes, detect a haemorrhagic event, identify specific surgical instruments, and to predict the next procedural step [8]. While there are many potential use cases, there are currently no widely used CV tools in clinical practice.

*Prediction of Intra-Operative Hypotension and Transfusion* The Hypotension Prediction Index (HPI) is an ML-derived early warning system, which has been extensively studied. Two RCTs performed in elective non-cardiac surgery patients found that the use of the HPI resulted in less intra-operative hypotension compared to standard care [29,30]. Notably, all studies were performed in patients undergoing elective surgery and their results cannot be generalised to trauma patients. Zapf et al. used an ML model to predict the need for intra-operative red blood cell (RBC) transfusion based on pre-operative variables [31]. The LightGBM model outperformed six other ML algorithms with an accuracy of 76.1% and sensitivity of 91.2% [31]. Predictive factors for RBC transfusion included the type of procedure, haemoglobin level, and was surgeon- and anaesthesiologist-dependent [31]. The use of individual surgeon and anaesthesiologist identifiers limits the generalisability of these results to other institutions. It also highlights the importance of training ML models with institution-specific or local data sets.

Post-operative

*Prediction of Survival and Complications* The power of AI algorithms lies in their ability to intelligently adjust the weight of input variables, capture non-linear relationships, and identify patterns in data sets. In contrast, traditional risk assessments such as the American Society of Anesthesiologists (ASA) score and Physiological and Operative Severity Score for the enUmeration of Mortality and Morbidity (POSSUM) often assume an additive and linear correlation [32]. The use of subjective scores like the ASA can result in misclassification, and conventional regression analysis used in POSSUM scores can over- or under-estimate risk, especially at extreme ranges of variables [33]. Novel smartphone-based AI risk prediction tools include Predictive Optimal Trees in Emergency Surgery Risk (POTTER), Trauma Outcome Predictor (TOP), and MySurgeryRisk [34-36]. POTTER uses a decision tree ML algorithm to predict post-operative morbidity and mortality after emergency surgery based on the American College of Surgeons (ACS) National Surgical Quality Improvement Program (NSQIP) data. POTTER was able to predict mortality and morbidity with great accuracy and surpassed surgeon gestalt [37,38]. POTTER’s c-statistic was 0.91 and outperformed ASA, Emergency Surgery Score, and the ACS-NSQIP calculator for predicting mortality [33]. TOP utilises optimal classification trees to predict mortality with an AUROC of 0.94 and 0.88 for penetrating and blunt injuries respectively, but it was less accurate at predicting complications such as acute kidney injury (AKI), pulmonary embolism, acute respiratory distress syndrome, deep venous thrombosis, and sepsis with an AUROC ranging between 0.69 to 0.84 [34]. MySurgeryRisk is based on a random forest model, which predicts 30- and 90-day mortality, as well as post-operative complications. It uses 135 input parameters and has an AUROC of 0.84 for predicting mortality [36]. MySurgeryRisk was better at predicting complications such as AKI, cardiovascular, delirium, prolonged mechanical ventilation, sepsis, wound, and venous thromboembolism compared to TOP with an AUROC ranging between 0.81 and 0.91 [36]. The platform display provides an output in real time and includes the risk of each post-operative complication, the top three risk factors for each complication, and patterns of complications for individual surgeons [36].

*Discharge Prediction* Understanding patient disposition for discharge can aid bed management and pre-emptive discharge planning. Kovoor et al. demonstrated that a ML-derived random forest model called the Adelaide Score can successfully predict discharge at 12 and 24 hours in general surgical patients with an AUROC of 0.85 and 0.84, respectively [39]. Input parameters included vital signs, pain scores, recent bowel movements, Charlson Comorbidity Index scores, and blood results, which were used to generate a score between 0 and 100, which represents a patient’s readiness for discharge following general surgery. The limitations of the Adelaide score include its lack of integration of socio-cultural factors in determining patient safety and readiness for discharge, and the requirement of an EMR system to make the score calculation practical for daily use [39].

Discussion 

Although there is great potential for AI in trauma systems, a multitude of challenges and risks exist. It will be imperative to have appropriate regulation, awareness of bias, and adequate education of the patient and the surgeon. National governance from external regulators, alongside the Royal Australian College of Surgeons, will be required to evaluate these novel AI tools to protect patients from profit-driven corporations or commercial entities [40]. AI technologies that are classified as Software as a Medical Device will need approval and regulation from the Therapeutic Goods Administration [41]. Additionally, revision of current malpractice guidelines will be required to define how responsibility is determined if both AI and human factors contribute to adverse events or outcomes [42].

Surgical decision-making often has time-sensitive and significant consequences, and the use of AI can introduce automation bias and safety concerns. Algorithmic outputs lack the nuances of human decision-making and require critical appraisal when applied to individual patient care. Clinicians should be cautious to prevent an over-reliance on AI-based tools. Other potential biases inherent in training data may contribute to or perpetuate existing inequalities in age, race, social-economic status, or sex, and must be scrutinised [43]. The performance of individual AI applications is heavily dependent on the characteristics, quality, and completeness of available datasets. The generalisability of outcome data may be limited by whether local or global datasets are used. The prevalence and incidence of blunt versus penetrating trauma, such as gunshot wounds, can vary significantly around the world. For example, AI models which have derived predictive, diagnostic, or decision-aid tools from databases from level I trauma centres in the United States may not be relevant to a regional trauma centre in Australia.

Finally, it is important to consider public opinion and attitude on the incorporation of AI into healthcare [40]. For many, AI is referred colloquially as a ‘black box’ with its inner workings obscure and difficult to explain. The clinician has a duty to counsel the patient about the factors considered by the AI decision-support tool and its limitations. Even amongst clinicians, it may be challenging to educate the patients on the conclusions or recommendations from AI tools outside of detailing the inputs and outputs of algorithms [44]. Clear policy is required to ensure patient privacy and data security. Moreover, patients need to be informed that their data could be retained or used for continued training of AI models, and opt-out options should be offered where possible [40].

## Conclusions

Despite the growing presence of AI in health care, many surgeons have a limited understanding about basic AI terminology and how these technologies work. This review highlights the multifaceted potential of AI to improve patient outcomes and streamline the pre-operative, intra-operative, and post-operative phases of thoraco-abdominal trauma care. Many trials have focused on assessing ML and DL in predictive analytics, diagnostics, and decision-making with promising results. There is evidence that these algorithms can complement, augment, and in some cases, surpass traditional risk prediction tools. However, current clinical applications of AI are limited. Surgeons should stay informed on the evolving role of AI so that it can be safely and effectively integrated into clinical care. Future prospective, locally validated research, rooted in Australian data sets, is required.
